# Posttraumatic stress symptom severity predicts cognitive decline beyond the effect of Alzheimer’s disease biomarkers in Veterans

**DOI:** 10.1038/s41398-023-02354-0

**Published:** 2023-03-29

**Authors:** Sarah Prieto, Kate E. Nolan, Jena N. Moody, Scott M. Hayes, Jasmeet P. Hayes, for the Department of Defense Alzheimer’s Disease Neuroimaging Initiative

**Affiliations:** 1 Department of Psychology, The Ohio State University, Columbus, OH USA; 2 Chronic Brain Injury Initiative, The Ohio State University, Columbus, OH USA

**Keywords:** Diagnostic markers, Psychiatric disorders

## Abstract

Chronic stress is a risk factor for dementia but whether it explains unique variance in cognitive decline in older adults above Alzheimer’s disease (AD) biomarkers is unknown. In a preclinical cohort of Vietnam Veterans, we examined the relationship between posttraumatic stress disorder (PTSD) symptom severity, AD biomarkers of beta-amyloid (Aβ) and tau, and change in cognitive performance on two widely-used screeners, the Mini-Mental State Examination (MMSE) and the Montreal Cognitive Assessment (MoCA). Analyses indicated that PTSD symptom severity was associated with a greater decline on the MMSE (*p* < 0.04) and MoCA (*p* < 0.024) after adjusting for biomarkers of AD, notably on the attention scale of the MoCA and the memory index of the MMSE. These analyses survived multiple comparison corrections. Taken together, PTSD symptom severity is associated with accelerated cognitive decline. Treating PTSD should be considered instrumental to maintaining cognitive function as adults age.

## Introduction

Posttraumatic stress disorder (PTSD) is a chronic, debilitating condition characterized by symptoms including intrusive re-experiencing of a traumatic event, avoidance of reminders of the event, negative mood/cognitions, and hyperarousal [1]. PTSD has been linked with poorer performance on neurocognitive tasks across various domains including attention, working memory, processing speed, verbal learning and memory, and executive functions [2–4]. There is growing concern that PTSD confers elevated risk for mild cognitive impairment and dementia [5, 6]. Studies have shown that patients with PTSD are twice as likely to develop dementia than those without PTSD [6], and a recent meta-analysis confirmed that PTSD is a strong risk factor for all-cause dementia [7]. The high prevalence of PTSD symptoms in populations such as military personnel underscores the need to understand the influence of PTSD symptoms on cognition in aging.

Although the mechanisms linking PTSD to Alzheimer’s disease (AD) and other dementias are unknown, there are several biological pathways that link stress with AD including neuroinflammation, metabolic disorders, and neuropathological markers such as beta-amyloid (Aβ) and tau [8–10]. Preclinical studies have shown that stress accelerates Aβ and tau pathology through corticotropin-releasing factor (CRF) overproduction [8, 9]. However, the results of human studies investigating the relationship between PTSD and AD biomarkers have been mixed. Three recent positron emission tomography (PET) studies found that PTSD did not increase risk of Aβ or total tau (t-tau) burden [11–13]. By contrast, another study using PET imaging found that PTSD was indeed correlated with Aβ burden [14]. Similarly, whereas one recent study found that PTSD did not increase risk of Aβ or tau burden using blood-based markers [12, 15], another study found decreased plasma Aβ in World Trade Center responders with PTSD [16]. Discrepant findings may be due to additional mechanistic contributors to observed associations between PTSD and cognitive decline. For example, researchers have posited that lifestyle factors including sleep disturbance, inflammatory responses of the immune system, decreased cognitive reserve, and metabolic and vascular diseases may contribute to the relationship between PTSD and dementia [17, 18]. Despite these discrepant findings, the potential to use these biomarkers to detect early onset of AD indicates that additional work is needed to clarify the relationship between PTSD, biomarkers of AD pathology, and cognitive outcomes, particularly in samples without known neurodegenerative disease. As such, an outstanding question remains whether the link between traumatic stress symptoms and cognitive decline is explained by elevated cerebrospinal fluid (CSF) AD biomarkers, or whether stress accounts for unique variance in cognitive decline among older adults [5, 19]. The substantial benefits of reliable biomarkers for early detection of neurodegenerative disease underscores the need for additional research in this area.

In the current study, we investigated associations between baseline PTSD symptom severity and longitudinal change in cognitive function using two widely used cognitive screeners in older adults, the Mini-Mental State Examination (MMSE) [20] and the Montreal Cognitive Assessment (MoCA) [21]. Both the MoCA and MMSE are frequently administered in clinical care settings and are advantageous given that they are quick, affordable, and can be administered in a broad range of settings [22, 23]. Data from the Vietnam Veterans Alzheimer’s Disease Neuroimaging Initiative Project (ADNI-DoD) was used. Given prior work implicating CSF Aβ42, t-tau, and phosphorylated tau (p-tau) in AD-related pathology, the association between PTSD symptom severity, AD biomarkers, and cognitive outcomes was examined. In addition, we examined history of military-related traumatic brain injury (TBI) as a covariate in analyses given that TBI may confer risk for development of PTSD, cognitive decline, and neurodegenerative disease [24, 25]. Moreover, there are high comorbidity rates of TBI and PTSD in Veteran samples, and PTSD/TBI comorbidity has been associated with increased neuroinflammation and greater cognitive dysfunction [26, 27].

## Material and methods

### Department of defense Alzheimer’s disease neuroimaging initiative database

Data used in the preparation of this article were obtained from the Brain Aging in Vietnam War Veterans/ Department of Defense Alzheimer’s Disease Neuroimaging Initiative (ADNI-DoD) database (adni.loni.usc.edu). ADNI-DoD is a nonrandomized, observational study launched in 2012 by Principal Investigator, Dr. Michael Weiner. This publicly available study recruited Veterans of the Vietnam war without dementia. The goal of ADNI-DoD is to understand the interplay between PTSD and TBI in influencing cognitive outcomes. For up-to-date information, see www.adni-info.org. Study procedures were approved by site-specific Institutional Review Boards and all participants and/or authorized representatives provided written informed consent. Data used in this manuscript were downloaded from the ADNI database (adni.loni.usc.edu) on April 6, 2021 from the following datasheets: CAPSCURR.csv, MoCA.csv, MMSE.csv, PTDEMOG.csv, UPENNBIOMK_DOD_2017.csv, and MEDHXSELF.csv. Data files are available upon request.

### Sample

ADNI-DoD recruited Veterans of the Vietnam War (1) with a history of moderate to severe non-penetrating TBI with or without PTSD, (2) with ongoing PTSD without TBI, and (3) without TBI or PTSD who are comparable in age, gender, education, and socioeconomic status. In addition, ADNI-DoD recruited Veterans without PTSD or TBI that were matched on age, sex, and education. Individuals were excluded from ADNI-DoD if they had a history of clinical evidence of stroke, a psychotic illness, or a neurologic illness. Moreover, potential participants were excluded for a diagnosis of mild cognitive impairment or dementia based on the Eight-item Informant Interview to Differentiate Age and Dementia [28] and Clinical Dementia Rating at baseline [29]. The full list of inclusion and exclusion criteria can be accessed on the online ADNI-DoD protocol (http://adni.loni.usc.edu/wp-content/uploads/2013/09/DOD-ADNI-IRB-Approved-Final-protocol-08072012.pdf).

Participants were included in the current analyses if they had demographic, PTSD, and CSF biomarker information available at baseline. To be included in this study, participants also had to have MoCA and MMSE scores at baseline and follow-up visits. If participants had information from more than one follow-up assessment available, then results from the most distant follow-up time from baseline were used to capture chronic cognitive changes. By not limiting our analyses to participants who had data available during a single timeframe, we were able to maximize the length of time between visits and the size of our sample.

### Posttraumatic stress severity

Current PTSD symptom severity at baseline was assessed with the CAPS-IV, in accordance with the DSM-IV-TR. This severity score was used because it captures individual variability in resilience and vulnerability to experiencing stressful events.

### Cognitive function

The MoCA and MMSE were used to measure cognitive function. Total scores were computed for performance on the MoCA and the MMSE for each participant. Scores ranged from 0 to 30. Replicating prior work, subdomains were calculated for the MoCA and MMSE [30–32].

#### Montreal Cognitive Assessment (MoCA)

MoCA scores were divided into six domains (orientation, language, visuospatial, attention, executive, and memory). The MoCA Orientation Index Score (MoCA-OIS) included the sum of points for all the Orientation items, with scores ranging from 0 to 6. The language index score (MoCA-LIS) included sums of scores for Naming, Sentence Repetition, and Letter Fluency, with scores ranging from 0 to 6. The visuospatial index score (MoCA-VIS) consisted of score sums from Cube Copy, Clock Drawing, and Naming, with scores ranging from 0 to 7. The Attention Index Score (MoCA-AIS) was calculated by adding the scores of Digit Span, Letter A Tapping, Serial 7 Subtraction, Sentence Repetition, and Words Recalled in Both Immediate Recall Trials, with scores ranging from 0 to 18 points. The Executive Index Score (MoCA-EIS) was calculated by adding scores for Trail-Making, Clock, Digit Span, Letter A Tapping, Serial 7 Subtraction, Letter Fluency, and Abstraction, with scores ranging from 0 to 13. The memory index score (MoCA-MIS) was calculated by adding the number of words remembered in the free delayed recall, category-cued recall, and multiple choice-cued recall, multiplied by 3, 2, and 1, respectively. Scores for the MoCA-MIS ranged from 0 to 15 points.

#### Mini-Mental State Examination (MMSE)

The MMSE was divided into four domains (orientation to time, orientation to place, working memory, and memory). The MMSE Orientation to Time Index Score (MMSE-OTIS) and the MMSE Orientation to Place Index Score (MMSE-OPIS) were sums of respective orientation items, with scores ranging from 0 to 5 points each. The MMSE Working Memory Index Score (MMSE-WMIS) was calculated by the score given to participants for spelling the word “WORLD” backwards. Scores ranged from 0 to 5 points. The MMSE Memory Score (MMSE-MIS) was the score for delayed recall of three words, with scores ranging from 0 to 3.

### Biomarkers

A subset of ADNI-DoD participants took part in a biomarker portion, where CSF was collected via lumbar puncture. CSF analyses were performed using Roche Elecsys immunoassay and following Roche Study protocol [33]. The Aβ42 assay has an established measurement range of 200–1700 pg/mL, while the t-tau assay has a measuring range from 80 to 1300 pg/mL, and the p-tau assay has a range from 8 to 120 pg/mL. Nineteen participants in our sample had Aβ42 values greater than the upper technical limit; these were truncated to 1700. No participants had values below the lower technical limit for Aβ42 or outside of the technical limits for t-tau (80–1300 pg/mL) or p-tau (8–120 pg/mL).

### Statistical approach

All data were analyzed using IBM SPSS Statistics for Macintosh, version 27 and R version 3.6.3 for Macintosh. Correlations were conducted between the length of time between cognitive measures (MoCA and MMSE) and variables of interest (i.e., age, years of education, CAPS-IV PTSD symptom severity scores, history of military-related TBI, Aβ42, t-tau, and p-tau).

All cognitive scores were standardized prior to analyses. Due to practice effects and other factors that may influence repeated cognitive assessments, a reliable change index (RCI) approach was implemented to calculate change for MoCA and MMSE total scores as well as for each subdomain using the following equation: ${\rm{RCI}} = \frac{{\left( {X_2 - X_1} \right) - \left( {M_2 - M_1} \right)}}{{\rm{S.E.D.}}}$, where X_1_ = individual participant’s standardized baseline score, X_2_ = individual participant’s standardized follow-up score, M_1_ = group mean baseline score, M_2_ = group mean follow-up score, and S.E.D. = standard error of difference score [34]. Cognitive performance was considered improved if the RCI resulted in a value greater than 1, while negative values were equivalent to a decline in cognitive performance.

To adjust for potential confounding effects, we examined whether there were significant associations between variables of interest and the length of time from follow up and baseline for either the MMSE and MoCA. Results revealed that there were no significant correlations between length of time from follow up to baseline for either the MMSE or MoCA and variables of interest, respectively (age: *p* = 0.170, *p* = 0.497; years of education: *p* = 0.884, *p* = 0.482; CAPS-IV severity scores: *p* = 0.964, *p* = 0.152; military-related TBI history: *p* = 0.055, *p* = 0.968; Aβ42: *p* = 0.892, *p* = 0.272; t-tau: *p* = 0.5650, *p* = 0.818; and p-tau: *p* = 0.860, *p* = 0.799).

Hierarchical linear regressions were conducted to examine PTSD symptom severity on cognitive performance over time. The dependent variable was the MoCA RCI. These procedures were replicated with the MMSE RCI. The following covariates were included in the first model: age at follow-up, education, history of TBI, and baseline cognitive performance. In the second model, Aβ42, t-tau, and p-tau were entered. The third model included PTSD symptom severity. Results were corrected for multiple comparisons of these two linear regressions (using change in MoCA and change in MMSE as dependent variables) using the Benjamini-Hochberg false discovery rate (FDR) procedure [35].

Follow-up analyses were conducted to determine the relationship between stress and change in the subdomains of each cognitive screener. As described above, covariates were entered into the first model, CSF biomarkers were entered into the second model, and PTSD symptom severity was entered into the third model. To account for multiple comparisons, the Benjamini–Hochberg’s critical procedure was implemented for the four subdomains of the MMSE and six subdomains of the MoCA.

In addition to using CSF biomarkers of AD, prior work has demonstrated the utility of PET imaging to quantify the presence of amyloid in the brain. Exploratory analyses were conducted to examine the agreement between CSF and PET metrics of AD biomarkers for a subsample of individuals who attended a PET scan visit. An amyloid PET florbetapir standardized uptake value ratio (SUVR) was calculated by averaging across cortical regions and dividing this cortical summary by the reference region consisting of the cerebellum, brainstem/pons, and subcortical white matter. For more information, please visit https://adni.loni.usc.edu/methods/documents. Higher SUVR reflects more amyloid deposition. Correlations were conducted between amyloid PET SUVR and CSF Aβ42, t-tau, and p-tau. Follow-up correlations were also conducted to determine the relationship between PET SUVR and change in cognition (MoCA RCI and MMSE RCI).

## Results

### Sample characteristics

Participant demographic and health characteristics are displayed in Tables 1 and 2. The sample used in the current analyses was restricted to males to avoid drawing invalid inferences, given the limited number of female participants available. As such, one female participant from the original sample was excluded from the current analysis. Thus, the final sample included 112 male participants. Most participants identified as White (83.9%). The timepoints from baseline to follow-up differed between participants, and times were chosen based on longest duration between visits as described in the “Methods” section. The number of months between baseline to follow-up ranged from 12 to 62 months (*M* = 23.79 months, SD = 14.42) for the MMSE and 9 to 51 months (*M* = 13.59 months, SD = 5.62) for the MoCA.

**Table 1:** Demographic characteristics (*N* = 112; 100% male).

Age at baseline years – Mean (SD)	68.5 (4.04)
Education years – Mean (SD)	15.2 (2.39)
CAPS-IV– Mean (SD)	30.7 (27.88)
Race – *n* (%)	--
White	94 (83.9%)
Black	6 (5.4%)
More than one race	4 (3.6%)
American Indian or Alaskan Native	3 (2.7%)
Other	5 (4.4%)
Hispanic – *n* (%)	12 (10.7%)

*CAPS-IV* Clinician-Administered PTSD Scale.

**Table 2:** Cognitive characteristics (*N* = 112; 100% male).

	Baseline visit	Follow-up visit
MoCA score	24.13 (2.893)	23.91 (3.411)
Orientation	5.81 (0.512)	5.78 (0.707)
Language	5.16 (0.865)	5.23 (0.943)
Visuospatial	6.15 (0.882)	6.02 (0.949)
Attention	16.33 (1.550)	16.13 (1.763)
Memory	9.46 (3.299)	9.27 (3.364)
Executive	11.42 (1.546)	11.31 (1.767)
MMSE score	28.31 (1.605)	28.19 (1.867)
Orientation to time	4.96 (0.207)	4.94 (0.278)
Orientation to place	4.64 (0.598)	4.68 (0.588)
Working memory	4.60 (0.895)	4.71 (0.693)
Memory	2.54 (0.709)	2.29 (0.907)

*MoCA* Montreal Cognitive Assessment; *MMSE* Mini-Mental State Examination. Note: The number of months between baseline to follow-up ranged from 12 to 62 months for the MMSE and 9 to 51 months for the MoCA.

### Associations between cognition, PTSD symptoms, and AD biomarkers

**Fig. 1:**
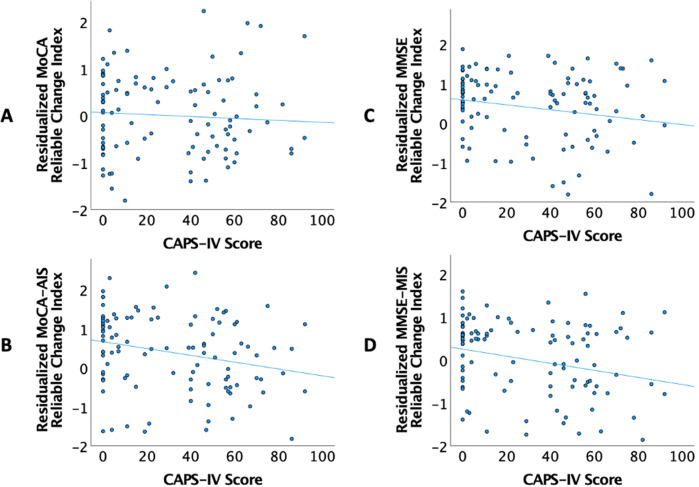
PTSD symptom severity and cognitive change. Higher scores on CAPS-IV were associated with greater decline in cognition. Values on the *x*-axis represent the CAPS-IV scores. Values on the *y*-axis represent standardized residuals of change in (**A**). MoCA total score, **B** attention index of the MoCA, and **C** MMSE total score, **D** memory index of the MMSE (accounting for age, education, history of TBI, baseline cognitive scores, Aβ42, t-tau, and p-tau). MoCA-AIS Attention Index Score of the MoCA; MMSE-MIS Memory Index Score of the MMSE; CAPS-IV Clinician-Administered PTSD Scale; Aβ42 Beta-amyloid; t-tau total tau; p-tau phosphorylated tau.

The association between PTSD symptom severity and change in the MoCA (RCI) was examined using a three-step hierarchical linear regression. In the first step, covariates (age at follow-up, education, baseline MoCA, and history of TBI) explained 16.8% of the variance, though only baseline MoCA was significantly associated with MoCA RCI. Results from the second model suggested that t-tau (*p* = 0.061), Aβ42 (*p* = 0.152), and p-tau (*p* = 0.156) were not significantly associated with MoCA RCI. Adding PTSD symptom severity to the model in step 3 revealed a significant effect. Specifically, higher PTSD symptom severity was associated with greater decline in MoCA scores after adjusting for age at follow-up, education, history of TBI, baseline MoCA scores, Aβ42, t-tau, and p-tau [Δ*F* (1,99) = 5.278, *p* = 0.024, Δ*R*^*2*^ = 0.037, *β* = −0.008; see Table 3 and Fig. 1A]. This corresponds to a partial *R*^2^ of 0.126. and to an effect size *f*^2^ = 0.144. This corresponds approximately to a medium effect size [36].

**Table 3:** Summary of regression analysis for association of CAPS-IV with MoCA change scores.

	Model 1				Model 2				Model 3			
Variable	*B*	SE	*β*	*P*	*B*	SE	*β*	*P*	*B*	SE	*β*	*P*
Age	−0.015	0.022	−0.061	0.509	−0.003	0.022	−0.014	0.88	−0.012	0.022	−0.051	0.58
Education	0.05	0.04	0.118	0.211	0.058	0.04	0.138	0.148	0.05	0.039	0.118	0.206
Baseline MoCA	−0.154	0.032	−0.441	<0.001**	−0.154	0.031	−0.439	<.001**	−0.175	0.032	−0.501	<.001**
TBI	0.175	0.184	0.084	0.345	0.166	0.179	0.08	0.357	0.096	0.178	0.046	0.590
Aβ42					0.000	0.000	0.155	0.152	0.000	0.000	0.175	0.099
T-tau					−0.011	0.006	−0.908	0.061	−0.009	0.006	−0.721	0.133
P-tau					0.08	0.056	0.668	0.156	0.055	0.056	0.456	0.330
CAPS-IV									−0.008	0.003	−0.222	0.024*
*R*^2^	0.199				0.263				0.300			
*F* change	6.405**				2.873*				5.278*			

*MoCA* Montreal Cognitive Assessment; *CAPS-IV* Clinician-Administered PTSD Scale; *TBI* Traumatic Brain Injury; *Aβ42* Beta-amyloid; Total Tau (t-tau); Phosphorylated Tau (p-tau),

*Significant at 0.05,

**Significant at 0.01.

The relationship between PTSD symptom severity and change in the MMSE was then examined using a three-step hierarchical regression. Covariates entered into the first model accounted for 28.5% of the variance, with baseline MMSE emerging as a significant predictor. When examining the contributions of biomarkers on change in MMSE in the second step, these biomarkers did not explain significant variance: Aβ42 (*p* = 0.505), t-tau (*p* = 0.893), and p-tau (*p* = 0.955). Adding PTSD symptom severity in step 3 revealed a significant effect, such that greater PTSD symptom severity was associated with decline in MMSE scores after adjusting for all covariates [Δ*F* (1,99) = 4.327, *p* = 0.04, Δ*R*^*2*^ = .03, *β* = −0.194; see Table 4 and Fig. 1C]. These results remained significant following correction for multiple comparisons. This corresponds to a partial *R*^2^ of 0.105. and to an effect size *f*^2^ = 0.117. This corresponds to a small to medium effect size [36].

**Table 4:** Summary of regression analysis for association of CAPS-IV with MMSE change scores.

	Model 1				Model 2				Model 3			
Variable	*B*	SE	*β*	*P*	*B*	SE	*β*	*P*	*B*	SE	*β*	*P*
Age	0.005	0.021	0.019	0.828	0.006	0.022	0.025	0.785	−0.002	0.022	−0.009	0.918
Education	0.028	0.037	0.065	0.456	0.035	0.039	0.083	0.368	0.025	0.038	0.059	0.514
Baseline MMSE	−0.346	0.055	−0.529	<0.001**	−0.347	0.057	−0.531	<0.001**	−0.37	0.057	−0.566	<0.001**
TBI	0.109	0.175	0.053	0.533	0.112	0.177	0.054	0.529	0.047	0.177	0.023	0.790
Aβ42					0.000	0.000	0.07	0.505	0.000	0.000	0.092	0.380
T-tau					−0.001	0.006	−0.063	0.893	0.001	0.006	0.081	0.863
P-tau					0.003	0.055	0.026	0.955	−0.017	0.055	−0.141	0.758
CAPS-IV									−0.007	0.003	−0.194	0.04*
*R*^2^	0.285				0.290				0.319			
*F* change	10.283**				0.196				4.327*			

*MMSE* Mini Mental State Exam; *CAPS-IV* Clinician-Administered PTSD Scale; Beta-amyloid (Aβ42); Total Tau (t-tau); Phosphorylated Tau (p-tau),

*Significant at 0.05,

**Significant at 0.01.

### Associations between cognitive domains and PTSD symptom severity

*MoCA*. Follow-up analyses were conducted to examine the association between PTSD symptom severity and change in domains of the MoCA. The MoCA attention index (MoCA-AIS) [Δ*F*(1,99) = 8.884, *p* = 0.004, Δ*R*^2^ = 0.057, *β* = −0.272; see Fig. 1B and Table 5] and MoCA orientation index (MoCA-OIS) were significant [Δ*F*(1,99) = 4.734, *p* = 0.032, ΔR^2^ = 0.026, *β* = −0.176]. After adjusting for multiple comparisons, the attention index (MoCA-AIS), but not the orientation index (MoCA-OIS), remained significant. The following domains of the MoCA were not significant: MoCA-MIS (*p* = 0.689), MoCA-EIS (*p* = 0.167), MoCA-VIS (*p* = 0.067), and MoCA-LIS (*p* = 0.053).

**Table 5:** Summary of regression analysis for association of CAPS-IV with MoCA attention reliable change index.

	Model 1				Model 2				Model 3			
Variable	*B*	SE	β	*P*	*B*	SE	β	*P*	*B*	SE	β	*P*
Age	−0.027	0.021	−0.112	0.201	−0.021	0.022	−0.085	0.340	−0.031	0.021	−0.125	0.153
Education	0.104	0.037	0.244	0.006	0.105	0.038	0.246	0.007	0.089	0.037	0.209	0.019
Baseline AIS	−0.313	0.055	−0.484	<0.001**	−0.311	0.056	−0.481	<0.001**	−0.354	0.055	−0.548	<0.001**
TBI	0.028	0.178	0.013	0.877	0.022	0.178	0.010	0.903	−0.077	0.175	−0.036	0.662
Aβ42					0.000	0.000	0.051	0.626	0.000	0.000	0.076	0.451
T-tau					−0.006	0.006	−0.476	0.311	−0.003	0.006	−0.239	0.601
P-tau					0.043	0.055	0.352	0.442	0.010	0.054	0.087	0.847
CAPS-IV									−0.010	0.003	−0.272	0.004*
*R*^2^	0.286				0.304				0.361			
*F* change	10.315**				0.845				8.884*			

*MoCA* Montreal Cognitive Assessment; *AIS* MoCA attention index; *CAPS-IV* Clinician-Administered PTSD Scale; Beta-amyloid (Aβ42); Total Tau (t-tau); Phosphorylated Tau (p-tau),

*Significant at 0.05,

**Significant at 0.01.

*MMSE*. Follow-up analyses were conducted to examine the association between PTSD symptom severity and change in domains of the MMSE. Results indicated that PTSD symptom severity explained significant variance in the memory index (MMSE-MIS) [Δ*F*(1,99) = 7.382, *p* = 0.008, Δ*R*^2^ = 0.045, *β* = −0.235; see Fig. 1D and Table 6]. This remained significant after adjusting for multiple comparisons. No other significant findings emerged (MMSE-OTIS *p* = 0.409; MMSE-OPIS *p* = 0.875; MMSE-WMIS *p* = 0.188).

**Table 6:** Summary of regression analysis for association of CAPS-IV with MMSE memory reliable change index.

	Model 1				Model 2				Model 3			
Variable	*B*	SE	*β*	*P*	*B*	SE	*β*	*P*	*B*	SE	*β*	*P*
Age	−0.011	0.024	−0.040	0.632	−0.005	0.024	−0.019	0.823	−0.018	0.024	−0.064	0.455
Education	0.033	0.041	0.067	0.426	0.029	0.043	0.059	0.502	0.013	0.042	0.027	0.752
Baseline MIS	−0.953	0.131	−0.583	<0.001**	−0.960	0.132	−0.587	<0.001**	−0.986	0.129	−0.603	<0.001**
TBI	−0.064	0.194	−0.026	0.744	−0.067	0.196	−0.027	0.734	−0.145	0.192	−0.060	0.451
Aβ42					0.000	0.000	−0.044	0.662	0.000	0.000	−0.015	0.875
T-tau					0.001	0.006	0.088	0.845	0.004	0.006	0.257	0.559
P-tau					−0.024	0.061	−0.171	0.695	−0.051	0.060	−0.361	0.400
CAPS-IV									−0.010	0.004	−0.235	0.008**
*R*^2^	0.342				0.351				0.396			
*F* change	13.370**				0.486				7.382**			

*MMSE* Mini Mental State Exam; *MIS* MMSE memory index; *CAPS-IV* Clinician-Administered PTSD Scale; Beta-amyloid (Aβ42); Total Tau (t-tau); Phosphorylated Tau (p-tau),

**Significant at 0.01.

### Exploratory analyses

Results revealed significant correlations between amyloid PET SUVR and Aβ42 *(*r = −0.431, *p* < 0.001), t-tau, (*r* = 0.361, *p* < 0.001), and p-tau: (*r* = 0.448, *p* < 0.001) in the expected directions. No significant associations emerged when examining the relationship between amyloid PET SUVR and the MoCA RCI (*r* = −0.130, *p* = 0.191) or the MMSE RCI (*r* = 0.069, *p* = 0.490).

## Discussion

This study examined the association between PTSD symptoms, AD biomarkers, and cognitive function over time. Specifically, we examined the relationship between PTSD symptom severity and two widely used brief screeners of cognitive function: the MMSE and the MoCA. Three main findings emerged. First, in a sample of Vietnam Veterans, PTSD symptom severity was associated with cognitive decline on both the MoCA and the MMSE. When examining particular domains of these cognitive screeners, we observed a relationship between PTSD symptom severity and changes in both (1) the attention domain of the MoCA and (2) the memory index of the MMSE. Third, our results indicate that PTSD symptom severity significantly predicted decline in cognition on both the screeners above and beyond what is explained by other potentially relevant factors (i.e., age, education, history of TBI, and baseline cognitive performance). Moreover, we did not observe relationships between AD biomarkers (i.e., Aβ42, p-tau, and t-tau) and change in cognitive function.

The findings reported here provide support for the notion that PTSD and symptom severity influences cognitive function and may accelerate cognitive decline [37, 38]. Specifically, the results corroborate cross-sectional studies demonstrating decreased cognitive performance among those with PTSD [39]. We extend previous work by examining these relationships longitudinally and considering the relative influence of AD biomarkers. These findings underscore the importance of considering traumatic stress when examining cognitive decline, however, it is important to acknowledge the effect size is relatively small, which is likely attributable to the short follow-up period.

Although the mechanism by which PTSD symptoms confer risk for cognitive decline is not yet fully understood, one possibility is stress-induced glucocorticoid dysregulation [40]. Animal models of stress have demonstrated that stress may engender a neurobiological cascade that begins with activation of the hypothalamic-pituitary-adrenal (HPA) axis, leading to dysregulation of cortisol production. Persistent elevations in cortisol result in the degradation of dendritic length and branches, inhibition of neurogenesis, and ultimately, reductions in synaptic plasticity [41]. Though there have been mixed findings, there is evidence that PTSD is associated with dysregulation in the HPA axis as evidenced through alterations in cortisol awakening response [42, 43] and enhanced cortisol suppression in response to dexamethasone [44]. Unexpectedly, some studies have found lower cortisol levels in a PTSD diagnosis compared to control groups. This may be explained by increased glucocorticoid receptor sensitivity due to overactivation of the HPA axis feedback loop [45, 46]. Although there are inconsistencies in the literature regarding the association between PTSD and the diurnal cortisol awakening response, it is generally understood that disruptions to HPA axis functionality is a key characteristic of PTSD [47].

In addition to glucocorticoid dysregulation, researchers have put forth other explanations for the link between PTSD and cognitive symptoms. For example, an alternative explanation relates to reduced cognitive and brain reserve being implicated in individuals with PTSD, as well as individuals with neurodegenerative diseases [18]. PTSD has also been associated with significant sleep impairments; for example, individuals with PTSD have been shown to have greater rapid eye movement (REM) sleep density and motor disturbances during REM sleep [48, 49], as well as reduced slow wave sleep. Sleep disturbances, in turn, may impact cognitive outcomes through hippocampal neurogenesis. Moreover, prior work has implicated sleep in glymphatic clearance of Aβ and tau [17, 50].

There is consistent evidence for anatomical differences in PTSD, including smaller hippocampal volume and cortical thinning in the medial prefrontal cortex [51–53]. Regions that have a high density of glucocorticoid receptors, such as the hippocampus and medial prefrontal cortex, have well-documented functions in memory functioning, including consolidation and retrieval of information [54]. In the current study, attention and memory were the most negatively affected by symptoms of PTSD. Our findings are consistent with prior work, which has shown that a PTSD diagnosis is associated with reduced cognitive function in the domains of attention, processing speed, and verbal memory [2]. These results have significant implications for public health, since they suggest that treating PTSD symptoms with evidence-based treatments may help preserve cognitive functioning in the context of aging. Bolstering resilience may be a potentially promising target to both prevent and treat symptoms of PTSD [55, 56]. Specifically, some interventions programs include adaptive coping, cognitive reappraisal, and mindfulness skills [57].

Previous work has shown that CSF biomarkers of AD, specifically Aβ42, t-tau, and p-tau, play a role in predicting change in cognitive function, even among samples without dementia [58, 59]. For example, prior work assessing World Trade Center responders identified increased AD plasma-based biomarker burden and linked these biomarkers with impaired cognition [16, 60]. In our study, we considered the possibility that baseline CSF biomarkers of AD, specifically Aβ, t-tau, and p-tau, play a role in predicting change in cognitive function. When examining the contributions of CSF biomarkers on change in cognitive function as assessed by the MMSE or MoCA, none of the biomarkers explained significant variance. One interpretation of these results is that amyloid- and tau-related neurodegeneration alone does not significantly contribute to cognitive decline. Taken together, results from this study suggest that PTSD symptom severity accounts for unique variance in cognitive functioning and may be a potent indicator of future decline in a non-demented sample. It is possible that longitudinal measurements of AD biomarkers would better predict changes in cognition in this sample of non-demented Veterans. Nevertheless, findings from the study suggest that PTSD symptom severity accounts for unique variance in cognitive function and may be a potent indicator of future decline in a non-demented sample. This is consistent with prior work showing PTSD was associated with a significant risk for dementia in both Veterans and the general population [7, 61].

The current findings suggest that PTSD symptoms are associated with cognitive decline as measured by widely-used cognitive screeners. The MMSE is the most commonly used measure of cognitive function [62, 63]. Though the MoCA is a newer assessment tool, it has gained traction among providers as a particularly effective tool at flagging cognitive dysfunction, particularly mild cognitive impairment [64]. Neurologists, primary care providers, and other medical professionals are trained to administer brief screening tools and can use information derived from these measures to determine whether further testing is warranted [62, 65]. Though these screeners should not be used for diagnostic purposes given their brevity and lack of diagnostic specificity, the current findings suggest that the screeners may be sensitive to the consequences of PTSD symptom severity.

This study has several limitations that should be noted. The study sample was composed primarily of White males, and thus may not generalize to a broader population. For example, a recent study demonstrated that in a sample of over 26,000 individuals, females had higher baseline cognitive functioning relative to men, but experienced faster cognitive decline in the context of aging [66]. As such, future work should strive to examine the relationship between PTSD and cognitive decline in females and a more diverse sample. Another important limitation of the current study relates to achieved power and small effect sizes due to our sample size. As such, future work should strive to replicate the current results in larger samples. In the current study, the average time between baseline and follow-up was an average of 23 months for the MMSE and 13 months for the MoCA. Future work should study these relationships over a longer time period. Additional work can be done to clarify the evolving relationship between PTSD, biomarkers of AD, and cognitive dysfunction longitudinally across different diagnostic categories (i.e., healthy controls, mild cognitive impairment, and dementia), as these relationships may change over the progression of the disease.

## Conclusion

*In conclusion*, results from this study clarify the relationship between PTSD and cognitive function in a sample of Vietnam Veterans. Specifically, our results suggest that PTSD symptom severity has been associated with decline in cognitive function in two frequently used cognitive screeners. These results were significant even after adjusting for the influence of biomarkers implicated in AD pathology. Moreover, PTSD symptom severity showed the greatest relationship with declines in memory and attention. These results underscore the impact of chronic stress on cognition in older adults and the importance of managing symptoms of chronic stress among older adults. Moreover, this information can help identify individuals who may benefit most from interventions meant to prevent AD. Additionally, these research findings may be used to help monitor individuals at risk and help inform treatment plans. Future work should address whether PTSD treatment can delay cognitive decline in older adults.
